# Left atrial appendage closure outcomes in relation to atrial fibrillation patterns: a comprehensive analysis

**DOI:** 10.3389/fcvm.2024.1389811

**Published:** 2024-05-22

**Authors:** Mingzhong Zhao, Jiangtao Yu, Cody R. Hou, Felix Post, Lei Zhang, Yuhui Xu, Nora Herold, Jens Walsleben

**Affiliations:** ^1^ Cardiovascular Center, Chengdu Shuangnan Hospital, Chengdu, China; ^2^ Heart Center, Zhengzhou Ninth People’s Hospital, Zhengzhou, China; ^3^ Department of Cardiology, Helmut-G.-Walther-Klinikum, Lichtenfels, Germany; ^4^ Clinic for General Internal Medicine and Cardiology, Catholic Medical Center Koblenz-Montabaur, Koblenz, Germany; ^5^ Cardiovascular Division, Department of Medicine, University of Minnesota Medical School, Minneapolis, MN, United States

**Keywords:** atrial fibrillation, atrial fibrillation pattern, left atrial appendage closure, outcomes, thromboembolism

## Abstract

**Objective:**

The effect of atrial fibrillation (AF) patterns on outcomes remains controversial. This study aims to evaluate the influence of AF type on the risk of cardiocerebrovascular events after left atrial appendage closure (LAAC) at long-term follow-up.

**Methods:**

AF was categorized as paroxysmal AF (PAF) and non-PAF (NPAF). The baseline characteristics, procedural data, peri-procedural complications, and long-term outcomes between patients with PAF and NPAF after LAAC were compared.

**Results:**

We analyzed 410 AF patients (mean age 74.8 ± 8.2 years; 271 male; 144 with PAF, 266 NPAF). The NPAF group tended to be older (≥75 years), male, and have chronic kidney disease (CKD) compared with the PAF group. The procedural data and peri-procedural complications were comparable. During 2.2 ± 1.5 years of follow-up, the incidences of thromboembolism, major bleeding, and device-related thrombus (DRT) did not differ between the two groups. The observed risk of thromboembolism and major bleeding was significantly lower than the estimated risk based on the CHA_2_DS_2_-VASc and HAS-BLED scores, respectively, in patients who underwent LAAC, regardless of the AF type. NPAF patients were associated with a higher risk of all-cause mortality, non-cardiovascular mortality, and combined efficacy endpoints. This association disappeared after propensity score matching (PSM) analysis.

**Conclusions:**

The risk of thromboembolism and major bleeding was lower in patients who underwent LAAC, regardless of the AF type. Although NPAF often coexists with multiple risk factors, it was not associated with worse long-term outcomes after LAAC when compared with PAF.

## 1 Introduction

Atrial fibrillation (AF) is the most frequent arrhythmia (1), of which non-valvular AF (NVAF) accounts for the majority of cases and is associated with 20%–30% of ischemic strokes (2). In clinical practice, AF is generally classified as paroxysmal AF (PAF) (i.e., AF episodes less than 7 days), persistent AF (i.e., AF episodes more than 7 days), long-standing persistent AF (i.e., continuous AF episodes >12 months), and permanent AF (i.e., no further attempts to restore sinus rhythm based on patient's and physician's desires) (3).

Due to the significant risk of cardiac embolism associated with NVAF, patients are recommended oral anticoagulants (OACs), including traditional OACs, such as warfarin, and direct oral anticoagulants (DOACs), such as dabigatran, rivaroxaban, apixaban, and edoxaban, to reduce this risk (4). In recent years, another non-pharmacological intervention strategy for the prevention of thromboembolism, known as percutaneous left atrial appendage closure (LAAC), emerged as an effective and safe alternative to OACs. LAAC has demonstrated non-inferiority in the prevention of stroke and less bleeding risk compared to OACs (5, 6).

Whether clinical outcomes vary among those with PAF vs. non-PAF (NPAF) has been an ongoing question. Thromboembolism risk stratification in AF primarily relies on patient-level risk factors instead of AF pattern (7). Interestingly, an increasing body of research evidence has demonstrated significant associations of AF patterns with clinical prognosis, which indicates an increased risk of thromboembolism and mortality in patients with NPAF compared to PAF receiving or not receiving oral anticoagulation (8, 9). However, research regarding the influence of AF types on long-term cardiovascular outcomes in patients who underwent LAAC is limited. Our study aims to evaluate the association of AF patterns with cardiovascular events in patients with LAAC.

## 2 Methods

### 2.1 Study population

We conducted a non-randomized, controlled retrospective study among NVAF patients who received LAAC consecutively with the WATCHMAN™ device (Boston Scientific, Marlborough, MA, USA) at Helmut-G.-Walther Klinikum, Lichtenfels, Germany, between February 2012 and June 2018, the WATCHMAN™ device and the LAmbre™ occluder (LifeTech Scientific Corp., Shenzhen, China) at Zhengzhou Ninth People's Hospital, Zhengzhou, China, from October 2016 to May 2022, and the WATCHMAN FLX™ occluder (Boston Scientific, Marlborough, MA, USA) at Chengdu Shuangnan Hospital, Chengdu, China, through July to October 2023. The inclusion criteria were as follows: NVAF patients aged more than 18 years; patients at increased risk of ischemic stroke, including those with CHA_2_DS_2_-VASc score ≥2 or a history of stroke, transient ischemic attack (TIA), or systemic embolism; patients with increased risk of major bleeding, such as those with HAS-BLED score ≥3 or a history of previous major bleeding or intolerance to long-term OAC treatment; or patients with preference for device implantation as a strategy of preventing thromboembolism. The exclusion criteria were a documented history of end-stage disease with less than 1 year of lifespan, the presence of intracardiac thrombus detected by imaging, or moderate to severe mitral valve stenosis. For each patient, written informed consent was obtained before the LAAC procedure. The study complies with the declaration of Helsinki and was approved by the institutional review boards of Helmut-G.-Walther Klinikum, Lichtenfels, Germany, Zhengzhou Ninth People's Hospital, Zhengzhou, China, and Chengdu Shuangnan Hospital, Chengdu, China.

### 2.2 LAAC procedure

The LAAC procedure was performed according to the vendor's implant protocol for the occluder. In general, transesophageal echocardiography (TEE) and cardiac computed tomography angiography (CTA) were used to assess the morphology and size of the LAA and exclude the presence of intracardiac thrombus before the procedure. Device implantation was performed under general anesthesia with the guidance of TEE and fluoroscopy imaging or under local anesthesia with the guidance of intracardiac echocardiography (ICE) and fluoroscopy imaging. After atrial septal puncture, heparin was intravenously administered to achieve an active clotting time of 250–350 s. The device was implanted at the LAA and released after meeting the criteria for size, positioning, stability of the device, and peri-device flow. An adequate closure of the LAA with good sealing and stability of the device and without device-related thrombus (DRT) was considered a successful device placement. The patients stayed in the hospital for 24–48 h and were discharged when no serious procedure-related complications were observed.

### 2.3 Antithrombotic strategy after the procedure

Antithrombotic drugs were required for a short period after LAAC to prevent thrombosis complications related to device implantation. In brief, OACs or aspirin plus clopidogrel were prescribed according to clinical situations during the first 45 days after LAAC. After this, all patients took aspirin plus clopidogrel until 6 months post-procedure if successful closure was confirmed by TEE imaging at the follow-up of 45 days post-procedure. At 6 months post-procedure, aspirin plus clopidogrel was replaced by aspirin alone indefinitely. Patients with unsuccessful closure, especially those with significant peri-device leak (>3 mm of peri-device leak) or presence of DRT, by TEE or cardiac CTA visit were initiated on OACs plus aspirin until good sealing of LAA or dissolution of thrombus identified by imaging.

### 2.4 Follow-up

Patient follow-up was performed by telephone or outpatient review at 45 days, 6 months after the procedure, and further visits every 6 months until the end of the study. A TEE evaluation was performed at 45 days, 6 months after the procedure, or a cardiac CTA scan was performed at 3 months after LAAC.

### 2.5 Endpoints

The endpoints of the study are as follows: (1) implantation success; (2) peri-procedural complications (ischemic stroke, TIA or systemic embolism, intracranial hemorrhage, other major bleeding, pericardial effusion/cardiac tamponade, severe access site complication, and procedure-associated death); and (3) long-term outcomes [thromboembolism (ischemic stroke, TIA, systemic embolism), major bleeding (intracranial hemorrhage, gastrointestinal bleeding, other major bleeding), DRT, all-cause mortality (cardiovascular mortality, non-cardiovascular mortality), and combined efficacy endpoints (thromboembolism and all-cause mortality)].

### 2.6 Statistical analysis

The categorical values are expressed as count (percentage). The continuous values are shown as mean ± standard deviation or median with interquartile ranges (IQR) (25th and 75th percentiles). The between-group differences were assessed by using Fisher's exact test for categorical values, Student's *t*-tests, or Mann–Whitney *U*-tests for continuous values.

The efficacy of LAAC on the risk of thromboembolism and major bleeding was assessed by comparing the difference between the observed risk and the predicted risk. The predicted risk of thromboembolism or major bleeding in a group was expressed as the mean percentage of each individually predicted annual risk (percentage) based on the CHA_2_DS_2_-VASc or HAS-BLED scores, respectively (10, 11). The observed risk of thromboembolism or major bleeding in a group was represented as the observed number of thromboembolism or major bleeding events per 100 patient-years, which originated from the numerical value of the total number of thromboembolism or major bleeding events divided by the total patient-years of follow-up, and then multiplied by 100. The relative risk reduction (RRR) in the events of thromboembolism and major bleeding was calculated as (predicted rate−observed rate)/predicted rate based on the Kaplan–Meier estimation. The Fisher's exact test, with relative risk (RR) and its 95% confidence intervals (CI), was used to assess the differences between the observed and predicted risks in each group and compare the discrepancies in the level of RRR between groups.

To decrease the influence of confounding factors on the mortality and combined efficacy endpoints, we performed a propensity score matching (PSM) analysis at a 1:1 ratio with a 0.02 propensity score tolerance to build the matched PAF and NPAF cohorts from the overall cohort. The patients between the PAF and NPAF groups were matched on variables that included age, gender, hypertension, coronary heart disease, diabetes mellitus, congestive heart failure, previous stroke, previous major bleeding, abnormal liver function, chronic kidney disease, CHA_2_DS_2_-VASc score, and HAS-BLED score. The Kaplan–Meier estimation was used to develop a time-dependent survival curve to evaluate the cumulative ratio of freedom from mortality or combined efficacy endpoints. The differences in the event curves between the matched groups were assessed via the log-rank (Mantel–Cox) test.

We used SPSS version 26.0 (SPSS Inc., Chicago, IL, USA) for analyzing data and GraphPad Prism software version 8.0 (GraphPad Software, LLC, San Diego, California, USA) for plotting a graph. A *P*-value of less than 0.05 (two-tailed) was deemed statistically significant.

## 3 Results

### 3.1 Patient demographics

Of the 420 patients with AF scheduled for the LAAC procedure, 410 patients successfully underwent LAA device implantation with a WATCHMAN™, LAmbre™, or WATCHMAN FLX™ occluder. LAAC was not performed in 10 patients (three cases in the PAF group and seven cases in the NPAF group) because of various contraindications, including unsuitable LAA morphology for WATCHMAN™ implantation in six cases, cardiac tamponade in two cases, repeated intra-procedural DRT formation in one case, and severe iliac vein stenosis in one case. Among the total cohort, 144 (35.1%) cases were categorized as PAF, while 266 (64.9%) cases were NPAF. Table 1 shows the patient baseline characteristics. The NPAF patients had a higher proportion of older patients (≥75 years) (61.6% vs. 51.3%, *P* = 0.047) and were more likely to be of male sex (70.6% vs. 57.6%, *P* = 0.009). In addition, the NPAF group was affected more often by chronic kidney disease (CKD) (45.5% vs. 34.7%, *P* = 0.036). The remaining baseline characteristics were comparable between the two groups (Table 1).

**Table 1:** Baseline characteristics of the enrolled patients.

Variables	Overall cohort	PAF group	NPAF group	*P*-value
	*n* = 410	*n* = 144	*n* = 266	
Age, years (mean ± SD)	74.8 ± 8.2	73.9 ± 8.3	75.2 ± 8.1	0.127
≥75 years, *n* (%)	238 (58.1)	74 (51.3)	164 (61.6)	0.047
Male, *n* (%)	271 (66.0)	83 (57.6)	188 (70.6)	0.009
Hypertension, *n* (%)	323 (78.7)	120 (83.3)	203 (76.3)	0.102
CHD, *n* (%)	210 (51.2)	70 (48.6)	140 (52.6)	0.469
Diabetes mellitus, *n* (%)	113 (27.5)	39 (27.0)	74 (27.8)	0.908
CHF^a^, *n* (%)	92 (22.4)	27 (18.7)	65 (24.4)	0.216
Previous stroke, *n* (%)	94 (22.9)	28 (19.4)	66 (24.8)	0.268
Previous major bleeding, *n* (%)	136 (33.1)	44 (30.5)	92 (34.5)	0.443
CKD^b^, *n* (%)	171 (41.7)	50 (34.7)	121 (45.5)	0.036
Abnormal liver function^c^, *n* (%)	49 (11.9)	14 (9.7)	35 (13.1)	0.342
CHA_2_DS_2_-VASc score (mean ± SD)	4.0 ± 1.6	3.9 ± 1.6	4.0 ± 1.5	0.601
HAS-BLED score (mean ± SD)	3.5 ± 1.1	3.4 ± 1.1	3.6 ± 1.0	1.000

Comparison of the baseline characteristics between patients with PAF and NPAF. PAF, paroxysmal atrial fibrillation; NPAF, non-paroxysmal atrial fibrillation; CHD, coronary heart disease; CHF, congestive heart failure; CKD, chronic kidney disease.

^a^ Defined as a left ventricular ejection fraction (LVEF) of <40% or the presence of CHF history.

^b^ Defined as an estimated glomerular filtration rate (eGFR) of <60 ml/min per 1.73 m^2^.

^c^ Defined as a history of prior liver disease or presence of elevated liver enzymes (alanine aminotransferase/aspartate aminotransferase ≥2× upper limit of normal) at admission.

### 3.2 Procedural characteristics

There was no significant difference in implantation success between the PAF and NPAF groups (98.0% vs. 97.4%, *P* = 1.000). In patients with NPAF, the ostium diameter and depth of the LAA were greater than those in patients with PAF. The overall rate of the peri-device leak was lower by 3.2%, similar for both groups. The remaining peri-procedural variables were comparable between groups (Table 2).

**Table 2:** Procedural data.

Variables	Overall cohort	PAF group	NPAF group	*P*-value
	*n* = 410	*n* = 144	*n* = 266	
LAA ostium diameter (mm)	20.5 ± 4.3	20.0 ± 3.9	20.8 ± 4.1	0.034
LAA depth (mm)	26.7 ± 5.1	25.8 ± 5.2	27.2 ± 5.4	0.012
Device implanted
Watchman, *n* (%)	391 (95.4)	134 (93.1)	257 (96.6)	0.138
LAmbre, *n* (%)	15 (3.7)	7 (4.0)	8 (3.0)	0.410
Watchman FLX, *n* (%)	4 (1.0)	3 (2.1)	1 (0.4)	0.127
Peri-device leak, *n* (%)	13 (3.2)	4 (2.8)	9 (3.4)	1.000
<3 mm, *n* (%)	12 (2.9)	4 (2.8)	8 (3.0)	1.000
3–5 mm, *n* (%)	1 (0.2)	0	1 (0.4)	1.000
>5 mm, *n* (%)	0	0	0	–
Contrast volume (ml), median (IQR)	80 (70, 100)	80 (70, 100)	90 (70, 102)	0.418
Fluoroscopy time (min), median (IQR)	8.4 (6.3, 11.9)	8.26 (6.1, 11.3)	8.66 (6.4, 12.3)	0.259
X ray-dose (mGy × cm^2^), median (IQR)	5,369 (3,236.6, 8,499.3)	5,375 (3,314.3, 7,870.5)	5,369 (3,194.5, 8,772.3)	0.625

Comparison of the procedural data between patients with PAF and NPAF. LAA, left atrial appendage; IQR, interquartile ranges.

### 3.3 Peri-procedural complications

Table 3 demonstrates the peri-procedural complications. A total of 14 (3.4%) patients had peri-procedural complications with five (3.5%) and nine (3.4%) cases in the PAF and NPAF groups, respectively. No significant differences were found between groups regarding the incidence of complications (Table 3).

**Table 3:** Peri-procedural complications.

Variables	Overall cohort	PAF group	NPAF group	*P*-value
	*n* = 410	*n* = 144	*n* = 266	
Ischemic stroke, *n* (%)	1 (0.2)	0	1 (0.4)	1.000
TIA, *n* (%)	0	0	0	–
Other systemic embolism, *n* (%)	0	0	0	–
Major bleeding, *n* (%)	2 (0.5)	1 (0.7)	1 (0.4)	1.000
Intracranial hemorrhage, *n* (%)	0	0	0	–
Other major bleeding, *n* (%)	2 (0.5)	1 (0.7)	1 (0.4)	1.000
Pericardial effusion/cardiac tamponade, *n* (%)	5 (1.2)	2 (1.4)	3 (1.1)	1.000
Severe access site complication, *n* (%)	6 (1.5)	2 (1.4)	4 (1.5)	1.000
Procedure-associated death, *n* (%)	0	0	0	–
Total, *n* (%)	14 (3.4)	5 (3.5)	9 (3.4)	1.000

Comparison of the peri-procedural complications between patients with PAF and NPAF. PAF, paroxysmal atrial fibrillation; NPAF, non-paroxysmal atrial fibrillation; TIA, transient ischemic attack.

### 3.4 Long-term outcomes

The average follow-up period was 2.2 ± 1.5 years, with a total of 893 patient-years, including 297 patient-years in the PAF group and 596 patient-years in the NPAF group. The follow-up length was comparable, and the risks of thromboembolism, major bleeding, and DRT were not statistically different between the two groups (Table 4). However, all-cause mortality and non-cardiovascular mortality were higher in the NPAF group compared with the PAF group (18% vs. 9%, *P* = 0.014; 10.5% vs. 3.4% *P* = 0.013), respectively. The combined efficacy endpoints were also significantly higher in patients with NPAF (21.4% vs. 10.4%, *P* = 0.006) (Table 4).

**Table 4:** Comparison of the long-term outcomes between patients with PAF and NPAF.

Variables	Overall cohort	PAF group	NPAF group	*P*-value
	*n* = 410	*n* = 144	*n* = 266	
Follow-up time, (days) (mean ± standard deviation)	794.6 ± 544.4	751.9 ± 562.2	817.7 ± 534.2	0.243
Thromboembolism, *n* (%)	14 (3.4)	2 (1.3)	12 (4.5)	0.152
Ischemic stroke, *n* (%)	9 (2.2)	1 (0.7)	8 (3.0)	0.169
TIA, *n* (%)	5 (1.3)	1 0.7)	4 (1.5)	0.661
Systemic embolism, *n* (%)	0	0	0	–
Major bleeding, *n* (%)	21 (5.1)	3 (2.0)	18 (6.7)	0.058
Intracranial hemorrhage, *n* (%)	3 (0.8)	0	3 (1.1)	0.555
GI bleeding, *n* (%)	16 (4.0)	3 (2.1)	13 (4.9)	0.191
Other major bleeding, *n* (%)	2 (0.5)	0	2 (0.8)	0.543
DRT, *n* (%)	20 (4.8)	6 (4.1)	14 (5.2)	0.811
All-cause mortality, *n* (%)	61 (14.8)	13 (9.0)	48 (18.0)	0.014
Cardiovascular mortality, *n* (%)	28 (6.8)	8 (5.5)	20 (7.5)	0.541
Non-cardiovascular mortality, *n* (%)	33 (8.0)	5 (3.4)	28 (10.5)	0.013
Combined efficacy endpoints, *n* (%)	72 (17.5)	15 (10.4)	57 (21.4)	0.006

PAF, paroxysmal atrial fibrillation; NPAF, non-paroxysmal atrial fibrillation; TEE, transesophageal echocardiography; TIA, transient ischemic attack; DRT, device-related thrombus; GI, gastrointestinal.

### 3.5 Effect of LAAC on thromboembolism and major bleeding by AF form

**Figure 1:**
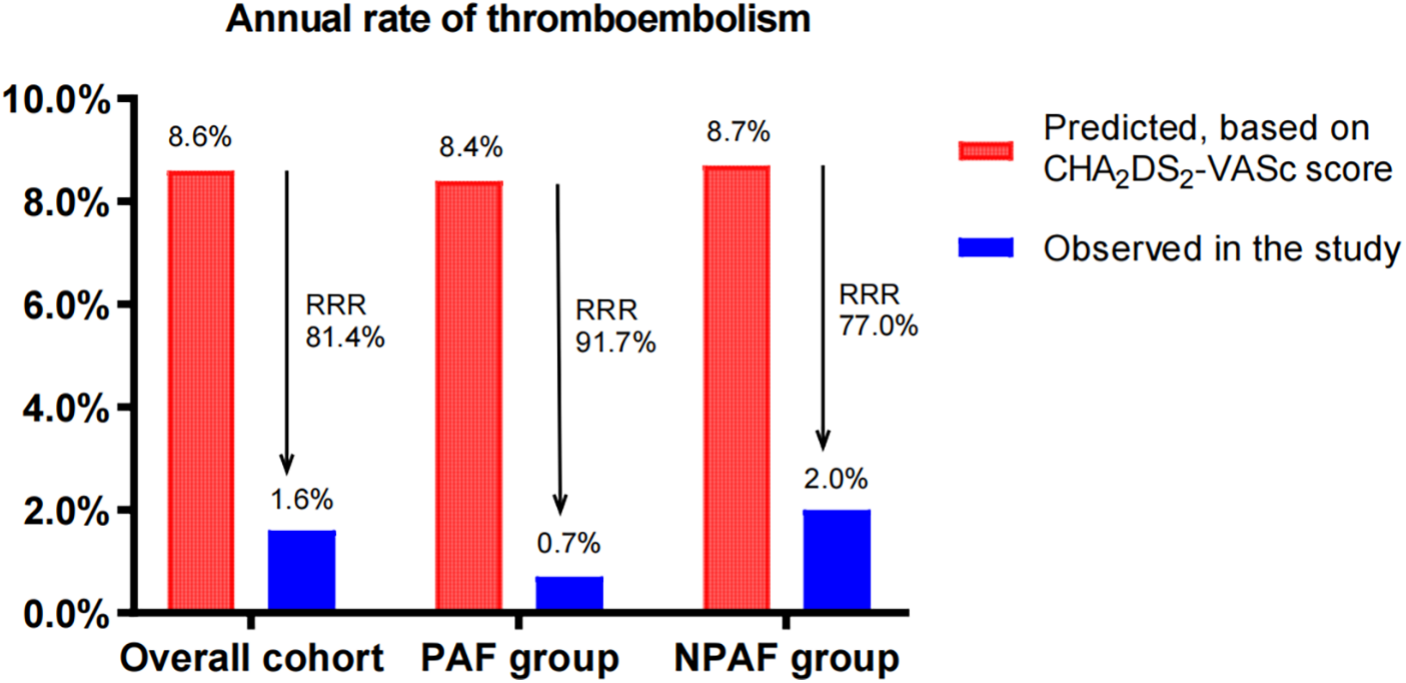
Effect of LAAC on thromboembolism in patients with PAF and NPAF. RRR, relative risk reduction; PAF, paroxysmal atrial fibrillation; NPAF, non-paroxysmal atrial fibrillation.

The cohort had a predicted thromboembolism risk of 8.6/100 patient-years (8.4/100 patient-years in the PAF group and 8.7/100 patient-years in the NPAF group). The observed thromboembolism rates were 1.6, 0.7, and 2.0/100 patient-years, yielding a relative risk reduction (RRR) of 81.4% (RR 5, 95% CI: 2.301–10.93, *P* < 0.0001), 91.7% (RR 12, 95% CI: 2.018–71.79, *P* = 0.0032) and 77.0% (RR 4.6, 95% CI: 1.832–11.61, *P* = 0.0007) in the overall cohort, PAF group, and NPAF group, respectively. Compared with the NPAF group, the PAF group had a greater extent of RRR (91.7% vs. 77.0%, RR 1.189, 95% CI: 1.093–1.293, *P* = 0.0001) (Figure 1).

**Figure 2:**
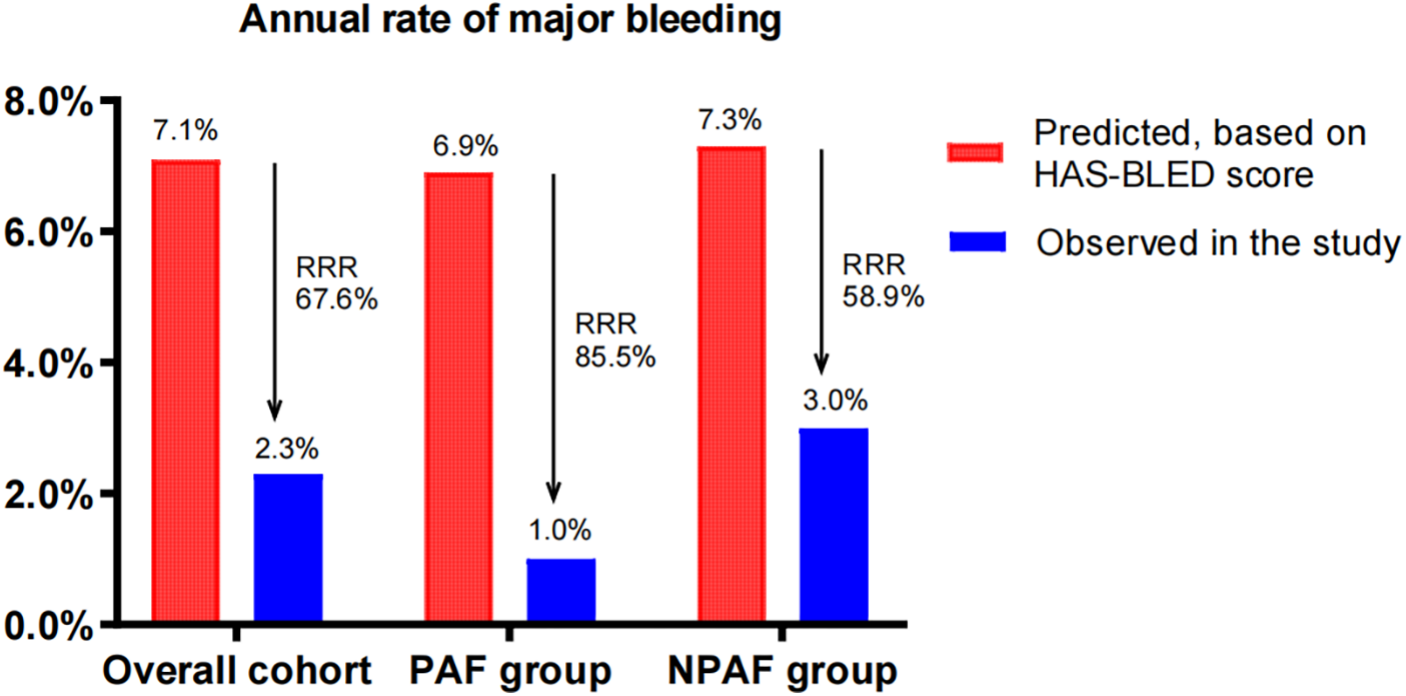
Effect of LAAC on major bleeding in patients with PAF and NPAF. RRR, relative risk reduction; PAF, paroxysmal atrial fibrillation; NPAF, non-paroxysmal atrial fibrillation.

The predicted annual major bleeding risks were 7.1, 6.9, and 7.3/100 patient-years in the overall cohort, PAF group, and NPAF group, respectively. However, the observed annual rates presented only 2.3, 1.0, and 3.0/100 patient-years, conferring an RRR of 67.6% (RR 3.222, 95% CI: 1.573–6.63, *P* = 0.0013), 85.5% (RR 10, 95% CI: 1.687–60.26, *P* = 0.0103), and 58.9% (RR 2.375, 95% CI: 1.102–5.172, *P* = 0.0415), respectively. The RRR of the PAF group was significantly greater than that of the NPAF group (85.5% vs. 58.9%, RR 1.447, 95% CI: 1.281–1.636, *P* ≤ 0.0001) (Figure 2).

### 3.6 Effect of LAAC on mortality and combined efficacy endpoints by AF form in the PSM cohort

**Figure 3:**
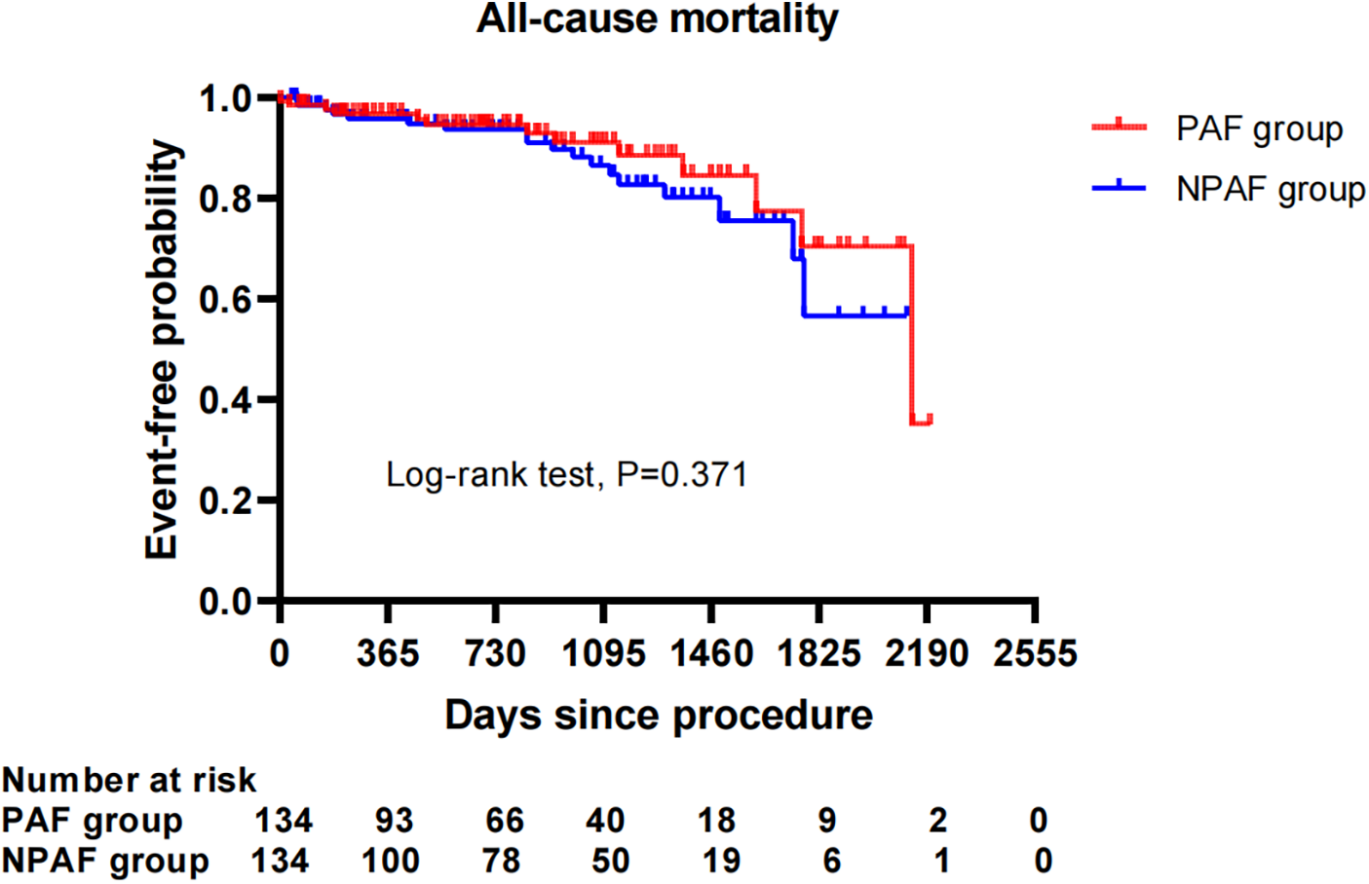
Comparison of the cumulative incidences of freedom from the all-cause mortality between the PAF and NPAF groups. PAF, paroxysmal atrial fibrillation; NPAF, non-paroxysmal atrial fibrillation.

**Figure 4:**
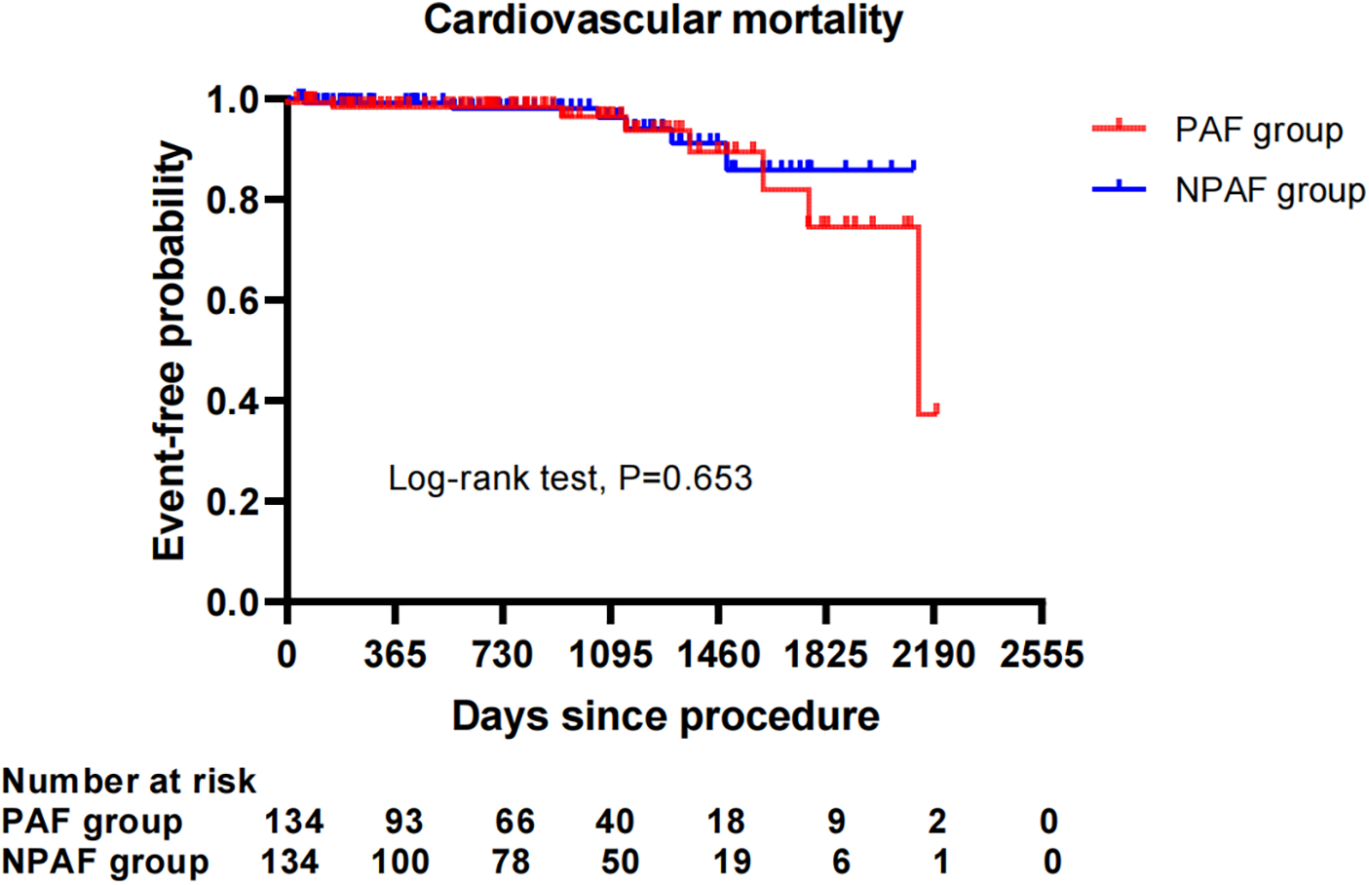
Comparison of the cumulative incidences of freedom from cardiovascular mortality between the PAF and NPAF groups. PAF, paroxysmal atrial fibrillation; NPAF, non-paroxysmal atrial fibrillation.

**Figure 5:**
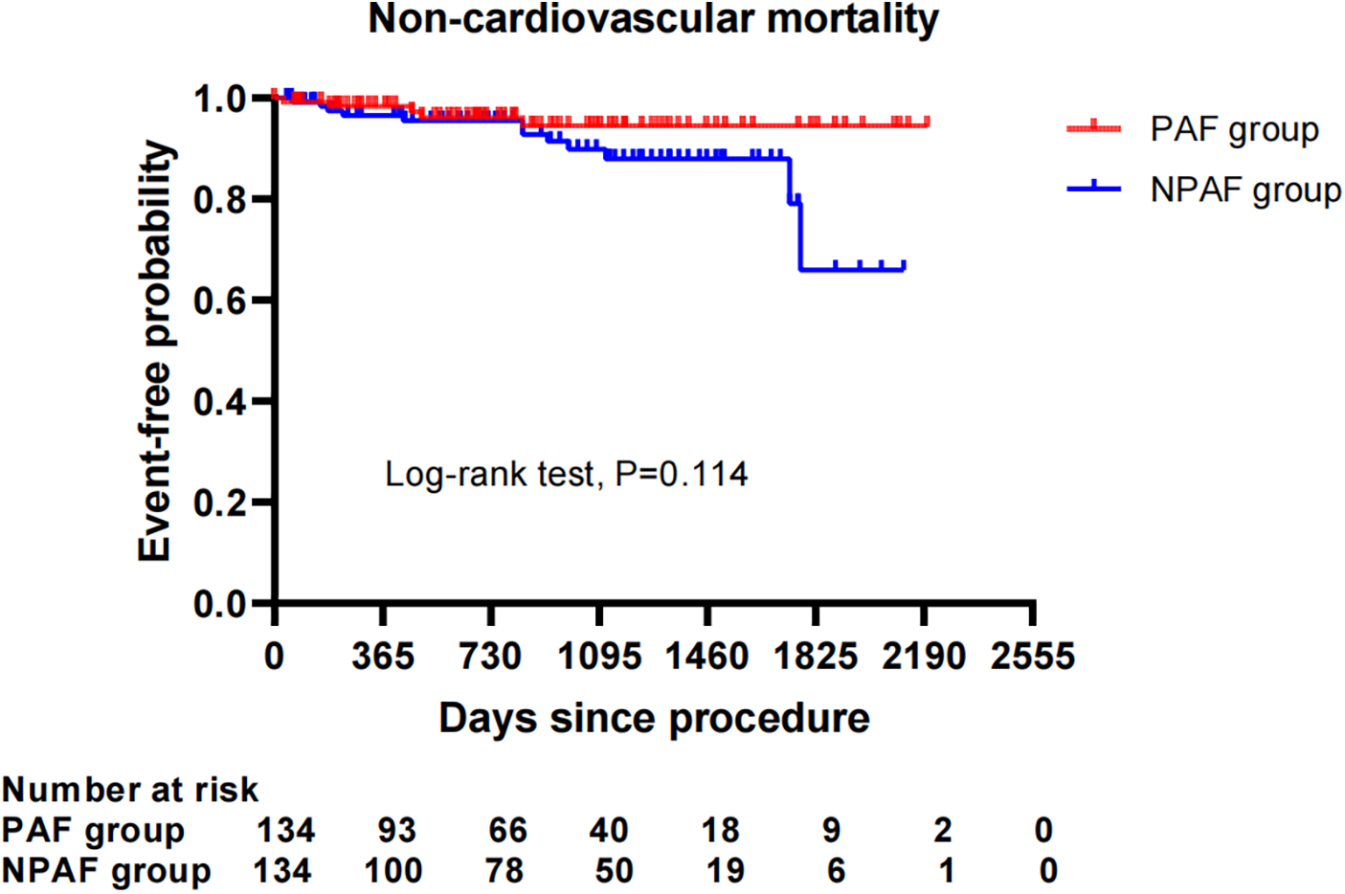
Comparison of the cumulative incidences of freedom from non-cardiovascular mortality between the PAF and NPAF groups. PAF, paroxysmal atrial fibrillation; NPAF, non-paroxysmal atrial fibrillation.

**Figure 6:**
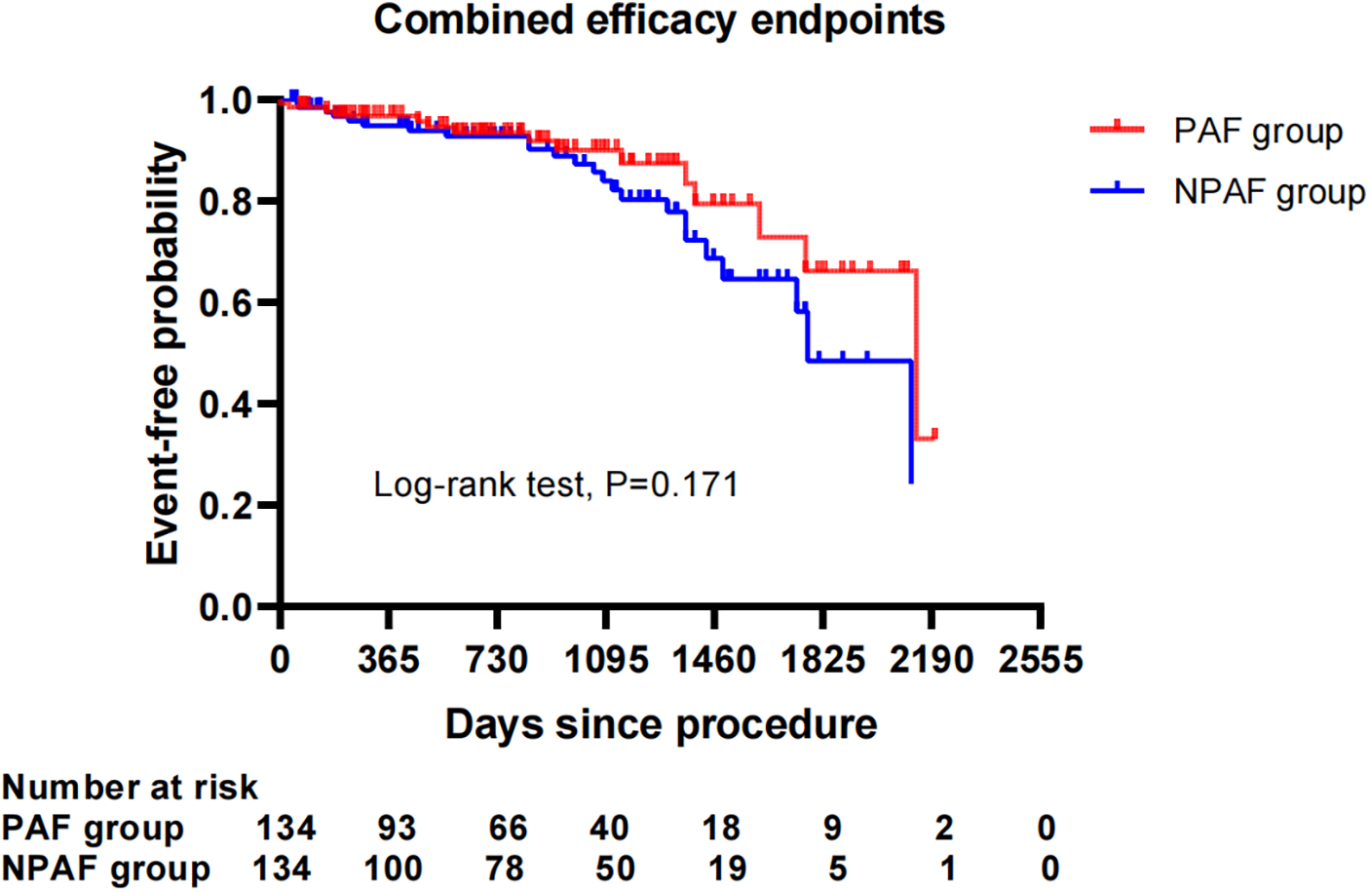
Comparison of the cumulative incidences of freedom from the combined efficacy endpoints between the PAF and NPAF groups. PAF, paroxysmal atrial fibrillation; NPAF, non-paroxysmal atrial fibrillation.

After performing the PSM analysis, a total of 268 patients formed a matched cohort, with 134 cases in the PAF group and 134 cases in the NPAF group and no differences in the baseline characteristics between groups (Table 5). The results from the Kaplan–Meier curves demonstrated that the cumulative ratio of freedom from all-cause mortality (*P* = 0.371) (Figure 3), cardiovascular mortality (*P* = 0.653) (Figure 4), non-cardiovascular mortality (*P* = 0.114) (Figure 5), or combined efficacy endpoints (*P* = 0.171) (Figure 6) did not differ between the two matched groups.

**Table 5:** Baseline characteristics of the PSM cohort.

Variables	Overall cohort	PAF group	NPAF group	*P*-value
	*n* = 268	*n* = 134	*n* = 134	
Age, years (mean ± SD)	75.0 ± 8.0	74.4 ± 8.3	75.5 ± 7.6	
≥75 years, *n* (%)	158 (59.0)	74 (55.2)	84 (62.6)	0.2637
Male, *n* (%)	163 (60.8)	83 (61.9)	80 (59.7)	0.8024
Hypertension, *n* (%)	233 (86.9)	115 (85.8)	118 (88.0)	0.7174
CHD, *n* (%)	134 (50.0)	67 (50.0)	67 (50.0)	1.000
Diabetes mellitus, *n* (%)	71 (26.4)	38 (28.3)	33 (24.6)	0.580
CHF^a^, *n* (%)	58 (21.6)	26 (19.4)	32 (23.8)	0.4585
Previous stroke, *n* (%)	63 (23.5)	27 (20.1)	36 (26.8)	0.249
Previous major bleeding, *n* (%)	90 (33.5)	40 (29.8)	50 (37.3)	0.2443
CKD^b^, *n* (%)	88 (32.8)	51 (38.0)	37 (27.6)	0.0906
Abnormal liver function^c^, *n* (%)	26 (9.7)	11 (8.2)	15 (11.1)	0.5365
CHA_2_DS_2_-VASc score (mean ± SD)	4.0 ± 1.6	4.0 ± 1.6	4.1 ± 1.5	0.5184
HAS-BLED score (mean ± SD)	3.4 ± 1.0	3.4 ± 1.1	3.4 ± 1.0	0.9011

Comparison of the baseline characteristics in the PSM cohort. PSM, propensity score matching; PAF, paroxysmal atrial fibrillation; NPAF, non-paroxysmal atrial fibrillation; CHD, coronary heart disease; CHF, congestive heart failure; CKD, chronic kidney disease; AF, atrial fibrillation.

^a^ Defined as a left ventricular ejection fraction (LVEF) of <40% or the presence of CHF history.

^b^ Defined as an estimated glomerular filtration rate (eGFR) of <60 ml/min per 1.73 m^2^.

^c^ Defined as a history of prior liver disease or presence of elevated liver enzymes (alanine aminotransferase/aspartate aminotransferase ≥2× upper limit of normal) at admission.

## 4 Discussion

Our principal findings are as follows: (1) LAAC provided a safe intervention strategy both in the PAF and NPAF patients; (2) no significant differences were found in the long-term risk of thromboembolism and major bleeding after LAAC between the two groups; LAAC significantly decreased the risk of thromboembolism and major bleeding when compared with the predicted risk, with a greater RRR in the PAF patients than in the NPAF patients; and (3) long-term follow-up showed that the incidence of all-cause mortality, non-cardiovascular mortality, and combined efficacy endpoints was significantly higher in patients with NPAF vs. PAF. However, these statistical differences no longer existed after adjustment for potential confounding factors.

Although current guidelines recommend that clinical decision-making on anticoagulation should be dependent on clinical risk stratification rather than specific AF type, there is increasing interest in evaluating if the burden of AF may provide an additional value in the prediction of the thromboembolic risk (12). A previous study showed that heart failure patients with paroxysmal AF were at a higher risk of heart failure hospitalization and stroke than those with NPAF due to more frequently used anticoagulants in the NPAF group (13). Antar et al. reported that in non-anticoagulated patients, NPAF significantly increased the risk of stroke/systemic embolism, worsening heart failure, and death compared with PAF. In anticoagulated patients, an increased risk of mortality, but not thromboembolism or worsening heart failure, remained in the NPAF patients (14). In PAF patients, a greater burden of AF was associated with an increased risk of ischemic stroke (15). How does the AF pattern influence the clinical outcomes in patients undergoing LAAC?

In this study, although the group with NPAF was older with a higher proportion of males and more likely to have CKD and a larger volume of LAA compared with the PAF group, the CHA_2_DS_2_-VASc and HAS-BLED scores were statistically similar. No significant differences were found in the peri-procedural complications between the two groups, implying that LAAC intervention had comparable safety for both the PAF and NPAF cohorts. LAAC is considered a complex procedure with worse peri-procedural outcomes, such as cardiac tamponade and severe peri-device leaks, in patients with higher risk factors. Recently, some studies reported that good imaging guidance, especially TEE or three-dimensional intracardiac ultrasound, could assist in the implementation of a complex LAA (16, 17).

LAAC, as a non-pharmacological alternative to anticoagulants, decreased the risk of stroke and major bleeding events in AF patients (18). This supported our findings on the observed thromboembolism and major bleeding risks being significantly reduced compared to the predicted risks across all AF patterns. Furthermore, our results still demonstrated that PAF patients experienced a greater risk reduction of thromboembolism and major bleeding when compared with NPAF patients after LAAC, implying that there are associations between the AF type and the extent of RRR in thromboembolism and major bleeding events by LAAC. However, our long-term follow-up did not show significant differences in the endpoint events of thromboembolism and major bleeding between the PAF and NPAF groups. This means that the AF type is not prognostic for the risk of thromboembolism and major bleeding in patients treated with LAAC.

The DRT incidence was reported to be approximately 3%–7% in patients following LAAC (19). Consistent with this finding, the DRT rate in our cohort was 4.8% without a significant difference in the AF pattern.

Previous studies showed that NPAF was significantly associated with higher risks of mortality and stroke/systemic embolism in patients without anticoagulation therapy (9, 20). NPAF was also associated with a higher mortality rate in those on anticoagulants after 1–2 years of follow-up (21). One “real-world” registry study also presented worse clinical outcomes in all-cause death in patients with NPAF vs. PAF, even while on oral anticoagulation (22). However, there are few studies evaluating the impact of the AF type on the prognosis in patients undergoing LAAC. Could the NPAF type affect long-term outcomes in patients following LAAC?

In our study with an average of 2.2 ± 1.5 years of follow-up, the incidences of all-cause mortality, non-cardiovascular mortality, and combined efficacy endpoints, instead of cardiovascular mortality, were significantly higher in NPAF vs. PAF patients after LAAC. These might be related to the higher risk profile in NPAF patients, including advanced age and higher incidence rate of CKD, which could worsen cardiovascular outcomes (23, 24). Interestingly, the cumulative ratio of freedom from all-cause mortality, non-cardiovascular mortality, and combined efficacy endpoints in the NPAF group were not significantly different from those in the PAF group after the PSM analysis was performed. This suggests that LAAC intervention might diminish the disparity in mortality and combined adverse endpoints resulting from the AF type. Recently, Kany et al. reported the effect of the AF pattern on outcomes following LAAC, showing that unadjusted mortality and combined endpoints of death, stroke, and systemic embolism significantly increased in NPAF patients compared with PAF patients at 1-year follow-up. Significantly higher combined endpoints of death, stroke, and systemic embolism remained after adjusting for potential confounding factors (25). The former was consistent with our findings, while the latter differed from our conclusions. The difference in the combined endpoints for the two PSM cohorts may be partially explained by an inconsistent period of follow-up with a longer follow-up duration in our cohort. Furthermore, future studies with a longer follow-up and a larger number of samples are needed to clarify the impact the AF type may have on adverse cardiocerebrovascular events after LAAC.

Our study has several limitations. This is an observational study without a randomized and controlled group; therefore, the power to evaluate the effect of the AF type on outcomes following LAAC may be limited. The AF pattern, especially PAF, may change during the long-term follow-up. In our analyses, we classified the AF group based on the initial AF pattern without monitoring the transformation of the AF type or the AF burden. Furthermore, not all patients underwent TEE investigation, which may underestimate the incidence of DRT or peri-device leak.

In conclusion, patients with NPAF tend to be older and have CKD. NPAF patients were not at higher risk of peri-procedural complications compared to those with PAF after LAAC. LAAC yielded favorable results in controlling thromboembolism and major bleeding, regardless of the AF type. The long-term outcomes following LAAC did not show differences in the AF pattern.

## Data Availability

The original contributions presented in the study are included in the article/Supplementary Material, further inquiries can be directed to the corresponding authors.

## References

[1] Wijesurendra RS Casadei B. Mechanisms of atrial fibrillation. Heart. (2019) 105(24):1860–7. 10.1136/heartjnl-2018-314267 31444267

[2] Boursier-Bossy V Zuber M Emmerich J. Ischemic stroke and non-valvular atrial fibrillation: when to introduce anticoagulant therapy? J Med Vasc. (2020) 45(2):72–80. 10.1016/j.jdmv.2020.01.153 32265018

[3] Hindricks G Potpara T Dagres N Arbelo E Bax JJ Blomström-Lundqvist C 2020 ESC guidelines for the diagnosis and management of atrial fibrillation developed in collaboration with the European Association For Cardio-Thoracic Surgery (EACTS): the task force for the diagnosis and management of atrial fibrillation of the European Society of Cardiology (ESC) developed with the special contribution of the European Heart Rhythm Association (EHRA) of the ESC. Eur Heart J. (2021) 42(5):373–498. 10.1093/eurheartj/ehaa612. 32860505

[4] Cheung CC Nattel S Macle L Andrade JG. Management of atrial fibrillation in 2021: an updated comparison of the current CCS/CHRS, ESC, and AHA/ACC/HRS guidelines. Can J Cardiol. (2021) 37(10):1607–18. 10.1016/j.cjca.2021.06.011 34186113

[5] Wintgens LIS Maarse M Swaans MJ Rensing BJWM Van Dijk VF Boersma LVA. The WATCHMAN left atrial appendage closure device for patients with atrial fibrillation: current status and future perspectives. Expert Rev Med Devices. (2020) 17(7):615–26. 10.1080/17434440.2020.1781615 32543911

[6] Osmancik P Herman D Neuzil P Hala P Taborsky M Kala P Left atrial appendage closure versus direct oral anticoagulants in high-risk patients with atrial fibrillation. J Am Coll Cardiol. (2020) 75(25):3122–35. 10.1016/j.jacc.2020.04.067 32586585

[7] January CT Wann LS Alpert JS Calkins H Cigarroa JE Cleveland JC Jr 2014 AHA/ACC/HRS guideline for the management of patients with atrial fibrillation. J Am Coll Cardiol. (2014) 64(21): e1–76. 10.1016/j.jacc.2014.03.022 24685669

[8] Steinberg BA Hellkamp AS Lokhnygina Y Patel MR Breithardt G Hankey GJ Higher risk of death and stroke in patients with persistent vs. paroxysmal atrial fibrillation: results from the ROCKET-AF trial. Eur Heart J. (2015) 36(5):288–96. 10.1093/eurheartj/ehu359 25209598 PMC4313363

[9] Ganesan AN Chew DP Hartshorne T Selvanayagam JB Aylward PE Sanders P The impact of atrial fibrillation type on the risk of thromboembolism, mortality, and bleeding: a systematic review and meta-analysis. Eur Heart J. (2016) 37(20):1591–602. 10.1093/eurheartj/ehw007 26888184

[10] Friberg L Rosenqvist M Lip GY. Evaluation of risk stratification schemes for ischaemic stroke and bleeding in 182 678 patients with atrial fibrillation: the Swedish atrial fibrillation cohort study. Eur Heart J. (2012) 33(12):1500–10. 10.1093/eurheartj/ehr488 22246443

[11] Lip GY Frison L Halperin JL Lane DA. Comparative validation of a novel risk score for predicting bleeding risk in anticoagulated patients with atrial fibrillation: the HAS-BLED (hypertension, abnormal renal/liver function, stroke, bleeding history or predisposition, labile INR, elderly, drugs/alcohol concomitantly) score. J Am Coll Cardiol. (2011) 57(2):173–80. 10.1016/j.jacc.2010.09.024 21111555

[12] Boriani G Vitolo M Diemberger I Proietti M Valenti AC Malavasi VL Optimizing indices of atrial fibrillation susceptibility and burden to evaluate atrial fibrillation severity, risk and outcomes. Cardiovasc Res. (2021) 117(7):1–21. 10.1093/cvr/cvab147 33913486 PMC8707734

[13] Mogensen UM Jhund PS Abraham WT Desai AS Dickstein K Packer M Type of atrial fibrillation and outcomes in patients with heart failure and reduced ejection fraction. J Am Coll Cardiol. (2017) 70(20):2490–500. 10.1016/j.jacc.2017.09.027 29145948

[14] Atar D Berge E Le Heuzey JY Virdone S Camm AJ Steffel J The association between patterns of atrial fibrillation, anticoagulation, and cardiovascular events. Europace. (2020) 22(2):195–204. 10.1093/europace/euz292 31747004 PMC7005596

[15] Go AS Reynolds K Yang JR Gupta N Lenane J Sung SH Association of burden of atrial fibrillation with risk of ischemic stroke in adults with paroxysmal atrial fibrillation: the KP-RHYTHM study. JAMA Cardiol. (2018) 3(7):601–8. 10.1001/jamacardio.2018.1176 29799942 PMC6145663

[16] Della Rocca DG Magnocavallo M Gianni C Mohanty S Al-Ahmad A Bassiouny M Three-dimensional intracardiac echocardiography for left atrial appendage sizing and percutaneous occlusion guidance. Europace. (2023) 26(1):euae010. 10.1093/europace/euae010 38225176 PMC10823354

[17] Pierucci N Gianni C Lawrence M La Fazia VM Natale A Kanj M Fourth time’s a charm: complex closure of incomplete left atrial appendage exclusion. JACC Cardiovasc Interv. (2024) 17(2):310–1. 10.1016/j.jcin.2023.07.011 37632481

[18] Schach C Reitschuster R Benedikt D Füssl E Debl K Maier LS Less major bleeding and higher hemoglobin after left atrial appendage closure in high-risk patients: data from a long-term, longitudinal, two-center observational study. Clin Cardiol. (2023) 46(11):1337–44. 10.1002/clc.24123 37573576 PMC10642336

[19] Garot P Cormier B Horvilleur J. Device-related thrombus after left atrial appendage closure. Interv Cardiol. (2019) 14(1):42–4. 10.15420/icr.2018.21.3 30858891 PMC6406133

[20] Ren J Yang Y Zhu J Wu S Wang J Zhang H Type of atrial fibrillation and outcomes in patients without oral anticoagulants. Clin Cardiol. (2021) 44(2):168–75. 10.1002/clc.23519 33314221 PMC7852164

[21] De With RR Marcos EG Dudink EAMP Spronk HM Crijns HJGM Rienstra M Atrial fibrillation progression risk factors and associated cardiovascular outcome in well phenotyped patients: data from the AF-RISK study. Europace. (2020) 22(3):352–60. 10.1093/europace/euz339 31865391

[22] Boriani G Laroche C Diemberger I Fantecchi E Popescu MI Rasmussen LH “Real-world” management and outcomes of patients with paroxysmal vs. non-paroxysmal atrial fibrillation in Europe: the EURObservational Research Programme-Atrial Fibrillation (EORP-AF) General Pilot Registry. Europace. (2016) 18(5):648–57. 10.1093/europace/euv390 26826133

[23] Kim D Yang PS You SC Jang E Yu HT Kim TH Age and outcomes of early rhythm control in patients with atrial fibrillation: nationwide cohort study. JACC Clin Electrophysiol. (2022) 8(5):619–32. 10.1016/j.jacep.2022.02.014 35589174

[24] Bansal N Zelnick LR Reynolds K Harrison TN Lee MS Singer DE Management of adults with newly diagnosed atrial fibrillation with and without CKD. J Am Soc Nephrol. (2022) 33(2):442–53. 10.1681/ASN.2021060744 34921110 PMC8819992

[25] Kany S Brachmann J Lewalter T Akin I Sievert H Zeymer U Impact of atrial fibrillation pattern on outcomes after left atrial appendage closure: lessons from the prospective LAARGE registry. Clin Res Cardiol. (2022) 111(5):511–21. 10.1007/s00392-021-01874-3 34043052 PMC9054864

